# Increase in Cellulose Accumulation and Improvement of Saccharification by Overexpression of Arabinofuranosidase in Rice

**DOI:** 10.1371/journal.pone.0078269

**Published:** 2013-11-04

**Authors:** Minako Sumiyoshi, Atsuko Nakamura, Hidemitsu Nakamura, Makoto Hakata, Hiroaki Ichikawa, Hirohiko Hirochika, Tadashi Ishii, Shinobu Satoh, Hiroaki Iwai

**Affiliations:** 1 Faculty of Life and Environmental Sciences, University of Tsukuba, Tsukuba, Ibaraki, Japan; 2 Division of Genome and Biodiversity Research, National Institute of Agrobiological Sciences, Tsukuba, Ibaraki, Japan; National Taiwan University, Taiwan

## Abstract

Cellulosic biomass is available for the production of biofuel, with saccharification of the cell wall being a key process. We investigated whether alteration of arabinoxylan, a major hemicellulose in monocots, causes an increase in saccharification efficiency. Arabinoxylans have β-1,4-D-xylopyranosyl backbones and 1,3- or 1,4-α-l-arabinofuranosyl residues linked to *O*-2 and/or *O*-3 of xylopyranosyl residues as side chains. Arabinose side chains interrupt the hydrogen bond between arabinoxylan and cellulose and carry an ester-linked feruloyl substituent. Arabinose side chains are the base point for diferuloyl cross-links and lignification. We analyzed rice plants overexpressing arabinofuranosidase (ARAF) to study the role of arabinose residues in the cell wall and their effects on saccharification. Arabinose content in the cell wall of transgenic rice plants overexpressing individual ARAF full-length cDNA (*OsARAF1*-FOX and *OsARAF3*-FOX) decreased 25% and 20% compared to the control and the amount of glucose increased by 28.2% and 34.2%, respectively. We studied modifications of cell wall polysaccharides at the cellular level by comparing histochemical cellulose staining patterns and immunolocalization patterns using antibodies raised against α-(1,5)-linked l-Ara (LM6) and β-(1,4)-linked d-Xyl (LM10 and LM11) residues. However, they showed no visible phenotype. Our results suggest that the balance between arabinoxylan and cellulose might maintain the cell wall network. Moreover, ARAF overexpression in rice effectively leads to an increase in cellulose accumulation and saccharification efficiency, which can be used to produce bioethanol.

## Introduction

Biofuel production from lignocellulosic biomass has attracted much attention as a method to reduce the consumption of fossil fuels, but a practical problem for use is that biofuel made from cellulosic biomass is costy because plant cell walls are very complex structures and are difficult to degrade. Therefore, for saccharification, the degradation of cell wall polysaccharides is a key process required to solve this problem.

Primary cell walls mainly consist of three polysaccharides: cellulose, hemicelluloses, and pectin. These polysaccharides determine the cell shape and mechanical strength. Lignin is a phenylpropanoid polymer deposited in the cell wall during secondary wall thickening [1]. Research on cell walls is important not only to understand mechanisms of plant growth and development but also to produce better plant material for practical use. Cell wall matrix polysaccharides generally occupy 10–30% of the dry weight of plants, and hemicellulose is responsible for cross-links between cellulose fibers. The dicot genus and monocot genus except for *Commelina* have type I cell walls and the hemicelluloses of members are rich in xyloglucan [2], while gramineous monocots have type II cell walls and their hemicellulose is rich in β-1,3-1,4-glucan and arabinoxylan. Arabinoxylan occupies 20–50% of the dry mass of the cell wall in primary and secondary cell walls [3] [4]. Arabinoxylans have a β-1,4-xylopyranosyl backbone and 1,3- or 1,4- α-l-arabinofuranosyl residues bonded at O-2 and/or O-3 of xylopyranosyl residues as side chains [2] [4] [5]. The xylan backbone has a 1,4-pyranose structure that is the same as cellulose or β-1,4-glucan, and forms strong hydrogen bonds with the surface of cellulose microfibrils. The arabinose side chain reportedly interrupts this hydrogen bond between arabinoxylan and cellulose, and the ratio of the addition of arabinose side chains to xylan backbone changes the wall mechanical properties [6]. Arabinose side chains can carry an ester-linked feruloyl substituent and these feruloyl groups form diferuloyl cross-links between arabinoxylans [7] [8], and in secondary cell walls, feruloyl acid is bonded to lignin polymers [9]. Thus, the arabinose side chain is the base point for diferuloyl cross-links and lignification. Although arabinofuranosyl residues are a quantifiably important constituent of plant primary and secondary cell walls, studies on this arabinose as a diferuloyl cross-link base point are lacking.

Genetic modifications of the cell wall have been reported [10], and plants with decreased hemicellulose and cellulose are generally physically weak and poorly adapted to the natural environment. For example, the cell wall network containing arabinose has been studied in dicots, and the loss of arabinose was found to be critical for plant development [11]. The *Arabidopsis* double mutant *rgp1rgp2* and transgenic UDP-arabinopyranose murase RNAi rice plants present lethal or dwarf phenotypes [12] [13]. In this paper, we focus on the functions of arabinose residues in arabinoxylan. We modified the arabinose content in rice using arabinofuranosidase (ARAF) overexpressor, Full-length cDNA overexpressor (FOX) lines [14] [15]. Using the endogenous enzyme may contribute to improved public acceptance of GM crops.

Beyond glycosyl composition analysis, we probed for wall modifications at the cellular level by comparing histochemical cellulose staining patterns and immunolocalization patterns using antibodies raised against α-(1,5)-linked l-Ara (LM6) and β-(1,4)-linked d-Xyl (LM10 and LM11) residues. We report the effect of a decrease in arabinose content by ARAF overexpression on maintenance of the cell wall network through arabinoxylan and cellulose and saccharification efficiency for production of bioethanol.

## Materials and Methods

### Plant material and growth conditions

Rice plants of the control (*Oryza sativa* cv. Nipponbare) and the two FOX lines AY311 and CO035, which carry overexpression constructs for *OsARAF1* (RAP locus: *07g0686900*) and *OsARAF3* (*Os11g0131900*), respectively, were grown in soil in a greenhouse during the natural growing season. In this environment, the temperature was 20–30°C and the light value was around 1,000 µmol s^–1^ m^–2^. All plants retained normal fertility.

The presence of the constructs in genomic DNA of these lines was confirmed by PCR. All experiments were performed with the T_3_ generation. Transgenic lines were selected on hygromycin-containing agar plates and tested for heritability of the expression pattern and the altered sugar trait.

### Phylogenetic analysis

BLAST searches were conducted with amino acid sequences of selected genes, namely *Oryza sativa* (members of GH family 51 and 3), *A. thaliana* (ARAF1, ARAF2, XLY1, and XLY3), and *H. vulgare* (AXHAI and AXAHII). A multiple alignment was generated by the neighbor-joining method in ClustalX [16] using full-length sequences and then manually adjusted. The phylogenetic tree was visualized using TreeView [17].

### RNA extraction and RT-PCR

Plant material was frozen in liquid nitrogen and ground with a Tissue Lyser II (Qiagen, Hilden, Germany). Total RNA was extracted using the RNeasy Plant Mini Kit (Qiagen, Hilden, Germany) and the DNase I recombinant (Roche, Basel, Switzerland) according to the manufacturers' protocols. cDNA was synthesized with ReverTra Ace® (Toyobo, Tokyo, Japan) according to the manufacturer's protocol. For the *OsARAF1*-FOX line, the quantity of transcripts was determined using primer sets of *OsARAF1*-forward (5′-CCAGAAGGGCAGTTATTG-3′) and *OsARAF1*-reverse (5′-CCAGAAGGGCAGTTATTG-3′). For the *OsARAF3*-FOX line, the quantity of transcripts was determined using primer sets of *OsARAF3*-forward (5′-GCTTCTCTTCCTTCCTTCCTTGT-3′) and *OsARAF3*-reverse (5′- CGATACTTGATCAAACCATACAACTCCCTT-3′). As an endogenous control, the quantity of 17S rRNA transcript were determined using primer sets of 17S rRNA-forward (5′-GCAAATTACCCAATCCTGAC-3′) and 17S rRNA-reverse (5′- CTATTGGAGCTGGAATTACC-3′). The amplified cDNA fragments were separated on 2% agarose gel and the bands stained with ethidium bromide.

### Enzyme assay

Enzyme assay in each line was measured according to [18]. Mature leaves were frozen in liquid nitrogen and ground with a Tissue Lyser II (Qiagen, Hilden, Germany). The following operations were carried out at 0–4°C. Ground samples were suspended in 20 mM sodium acetate buffer (pH 5.0) containing 1 M sodium chloride for 2 h. After centrifugation at 10,000× *g* for 5 min, the supernatant was applied to a PD-10 column midi-Trap G-25 (GE Healthcare, Milwaukee, WI, USA) and the eluted fraction was used for the enzyme assay. The concentration of protein was determined by the method of Bradford, with bovine serum albumin as the standard [19]. Enzyme activities were determined using a reaction mixture (200 µl) consisting of protein fractions, 25 mM acetate buffer (pH 5.0), and 1 mM *p*-nitrophenol glycoside. After incubation at 37°C for 2 h, the reaction was terminated by the addition of 200 mM sodium carbonate (800 µl) and monitored at 420 nm. One unit of enzyme activity is defined as 1 µmol of *p*-nitrophenol liberated per minute at 37°C min^–1^.

### Extraction and analysis of cell wall polysaccharides

Mature leaves of FOX lines were frozen in liquid nitrogen and ground with a Tissue Lyser II (Qiagen, Hilden, Germany) at 30 Hz for 2 min, and the resulted powder washed in 80%(v/v) EtOH. The supernatant was removed after centrifugation for 5 min at 15,000× *g*. The pellet was washed three times with water, three times with methanol:chloroform (MC = 1∶1v/v), and three times with acetone. A drop of phenol:acetic acid:water (PAW = 2∶1∶1v/v) was added to the pellet and mixed. Two drops of MC were added to the sample and washed with acetone. This process was repeated three times and the sample was then dried at room temperature for over 1 h. Starch was removed by digestion with amylase (2 unit/ml amylase; Wako, Osaka, Japan) in 50 mM acetate buffer at 37°C for 3 h. After reaction, the samples were centrifuged and the residues washed three times with water, MC, and acetone. After washing, the samples were air-dried for over 12 h. Alcohol-insoluble residues (AIRs) were used as the cell wall material. A total of 2 mg of AIR was hydrolyzed with 2 M trifluoroacetic acid (TFA) at 121°C for 2 h. After hydrolysis, the samples were centrifuged at 15,000× *g* for 5 min. The supernatant was the TFA-soluble fraction. The pellets were hydrolyzed with 72% H_2_SO_4_ at room temperature for 2 h and then diluted to 4% H_2_SO_4_ and boiled for 1 h. The H_2_SO_4_ solutions were neutralized with Ba(OH)_2_. Sugar in TFA-soluble and -insoluble fractions was treated with methanol:hydrogen chloride and the resulting methyl glycosides were converted into trimethylsilyl (TMS) derivatives and analyzed by gas-liquid chromatography (GC-14; SHIMADZU Kyoto, Japan). Sugar content in TFA-soluble and TFA-insoluble fractions was determined using the phenol sulfuric acid method.

### Cellulose analysis

Crystalline cellulose was measured according to [20]. Briefly the samples were treated with acetic and nitric acids to remove non-cellulosic polysaccharides, and the remaining pellets were hydrolyzed with 72% sulfuric acid. Glucose content in sulfuric acid was determined by phenol sulfuric acid method.

### Lignin measurement

Lignin contents in each line were measured according to [21]. Explaining briefly, mature leaves were frozen in liquid nitrogen and ground with a Tissue Lyser II (Qiagen, Hilden, Germany) at 30 Hz for 2 min. 3N HCl and 0.1 ml thioglycolic acid were added to 20 mg of AIR and heated at 80°C for 3 hours. After centrifugation, the pellet was dissolved in 1N NaOH. The solution was submitted to spectrophotomeric measurement.

### *p*CA, FA and DFA measurement

Cell wall was treated with 1N NaOH for 12h, and acidified with HCl. *p*CA, FA and DFA liberated were extracted three time with diethyl ether. The extract was air-dried, then stored in the dark until determination. *p*CA, FA and DFA contents were determined using HPLC system (Shimadzu, Kyoto, Japan) with a column Luna C18(2) column, (150×4.60mm, Phenomenex, USA) and monitored for UV(262 nm) and fluorescence (330 and 435 nm). The column equilibrated with 5 mM ammonium acetate (pH 4.4) containing 25% methanol was eluted by a linear gradient of methanol (25–50%, 2–10 min) and 5 mM ammonium acetate (pH 4.4) containing 50% methanol (10–20 min) at flow rate of 1.0 ml/min.

### Immunohistochemistry and cellulose staining

To perform immunolocalization, leaves of the control, *OsARAF1*-FOX, and *OsARAF3*-FOX lines at the same developmental stage were fixed with 4% paraformaldehyde, 0.25% glutaraldehyde, and 0.05 M phosphate buffer (pH 7.5) and embedded in 5% agar. Sections of 30 µm were cut with a microtome (VT1200S; Leica Microsystems, Nussloch, Germany). The TSATM Kit no. 12, with HRP-goat anti-rabbit IgG and Alexa Fluor 488 tyramide (Molecular Probes/Invitrogen, Eugene, OR, USA), was used according to the manufacturer's protocol. The primary antibodies LM6, LM10, and LM11 (PlantProbes, Leeds, UK) were used at a 1∶30 dilution. HRP conjugate working solution was used at a 1∶100 dilution. A negative control experiment was performed without the primary antibody. Cellulose was stained with 0.01% calcofluor white (Fluorescent Brightener 28; Sigma-Aldrich, St. Louis, MO, USA). All sections were observed using fluorescence microscopy (Leica Microsystems, Wetzlar, Germany), with UV filter A, excitation filter BP 340–380 nm, suppression filter LP 425, and an exposure time of 190 ms.

### Measurement of mechanical properties

Mature leaves of 7–8 mm width of the control, OsARAF1-FOX, and OsARAF3-FOX were cut 12 cm from the leaf tip and immediately used to assess mechanical properties. Both sides of a sample were adhered to the stage, with an interval of 2 cm; the stage moved to load the middle of the sample on the sensor; and then the change of load (break force) and extension length per leaf width were monitored using EZ Graph (Shimadzu, Kyoto, Japan).

### Measurement of saccharification efficiency

Mature leaves were ground with a Tissue Lyser II (Qiagen, Hilden, Germany) at 30 Hz for 2 min. An aliquot of 1.2 ml of 0.1 M sodium acetate buffer (pH 4.5) was added to 40 mg of the resulting powder and suspended. A sample of 60 µl was recovered and 40 µl sodium acetate buffer added at time 0. A total of 10 mg/ml Meiselase (Meiji, Tokyo, Japan) was added to the remaining sample and reacted at 45°C with shaking at 120 Hz. After 1–24 h, 100 µl suspensions were recovered followed by centrifugation at 15,000× *g* for 10 min at room temperature. Sugar content in the supernatant was determined by the phenol sulfuric acid method. The saccharification efficiency was calculated as sugar liberation (%)  =  µg/mg dry weight of leaves.

## Results

### Selection of ARAF genes from the FOX library

**Figure 1:**
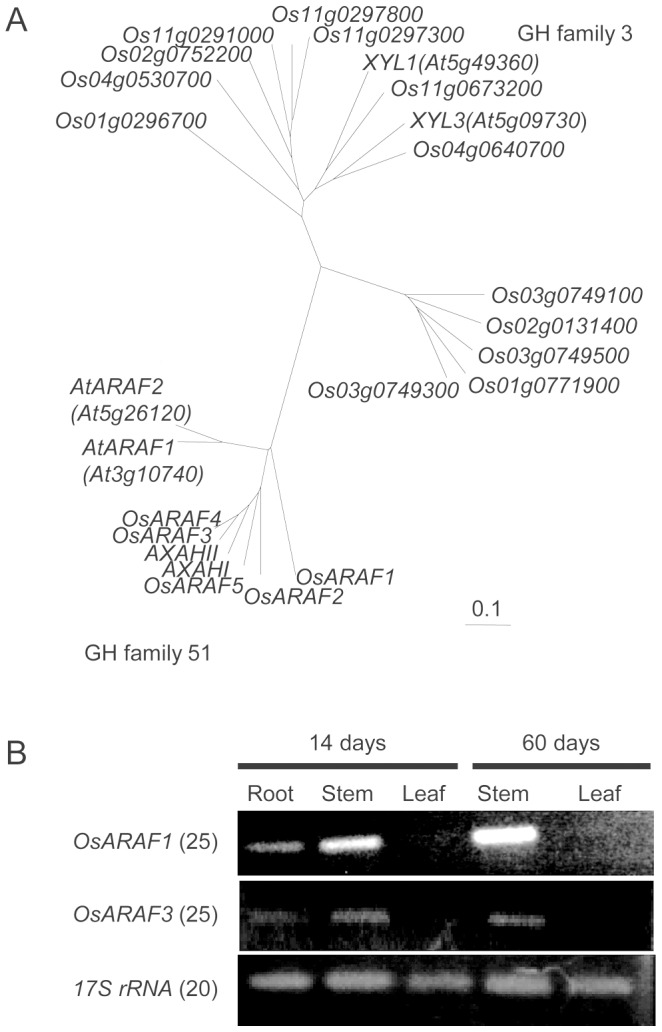
Characteristics of *Oryza sativa ARAF1* and *ARAF3*. (A) Phylogenetic tree of putative ARAFs and xylosidases (members of the GH families 51 and 3) in *Oryza sativa*, Arabidopsis *thaliana* (*AtARAF1*: *At3g10740*, *AtARAF2*: *At5g26120*, *XLY1*: *At5g49360*, *XYL3*: *At5g09730*) and *Hordeum vulgare* (*AXHAI* and *AXAHII*). Phylogenetic trees were constructed by the neighbor-joining method in ClustalX. (B) The expression patterns of *OsARAF1* and *OsARAF3*. RT-PCR analysis was performed using total RNA isolated from different organs of 14-day-old seedlings and 60-day-old mature plants. The numbers in parentheses indicate the numbers of PCR cycles. These experiments were performed at least twice with similar results.

To select the ARAF genes of rice, we searched the Rice PIPELINE database (http://cdna01.dna.affrc.go.jp/PIPE/; [22]) and identified 16 putative ARAF genes with reference to the Carbohydrate-Active enzyme [CAZy] database (http://www.cazy.org; [23] [24]), showing that the ARAF genes are members of GH family 3 and 51. We generated a phylogenic tree using 16 rice *ARAF* genes, four *Arabidopsis thaliana* genes (*AtARAF1*: *At3g10740*, *AtARAF2*: *At5g26120*, *XYL1*: *At5g49360*, *XYL3*: *At5g09730*), and two *Hordeum vulgare* genes (*AXHAI* and *AXAHII*). Several enzymes of the GH family 3 and 51 have been reported to have bifunctional activity for ARAF/β-d-xylosidase. *Arabidopsis* XLY1 and XLY3 [25] [26] have activity to hydrolyze *p*-nitrophenyl-α-l-arabinofuranoside (PNP-ARAf), *p*-nitrophenyl-β-d-xylopyranoside (PNP-Xyl), oat spelt xylan, rye arabinoxylan, wheat arabinoxylan, and oligo-arabinoxylan [25]. ASD1 and ASD2 have only ARAF activity [27]. The barley AXAH-I and AXAH-II have ARAF activity to release arabinose from 1,5-α-l-arabinopentaose, sugar beet arabinan, wheat arabinoxylan, and larchwood (*Larix*) arabinogalactan, but do not release d-Xyl [28] [29]. The phylogenetic tree showed that enzymes belonging to GH family 3 have bifunctional activity for ARAF/β-d-xylosidase and those in GH family 51 seemed to only have ARAF activity (Figure 1A). Therefore, we selected genes from GH family 51 for further study.

For a systematic approach to analyze a gain-of-function phenotype, [14] [15] developed the FOX hunting system by using expression libraries for full-length cDNAs (fl-cDNAs) from rice at a maximum of 28,000 fl-cDNAs clones in total, and individually overexpressed the fl-cDNAs in rice driven by the maize *Ubiquitin-1* gene promoter. Amoung ∼14,500 FOX-rice lines, we identified two lines overexpressing fl-cDNAs for OsARAF1 (Accession no. AK064838, Rap-ID:Os07g0686900), and OsARAF3 (AK065240, Os11g0131900) in GH family 51 and named them the *OsARAF1*-FOX and *OsARAF3*-FOX lines. We analyzed a total of 23 plants from the T1 to T3 generations; they showed essentially identical results. Since OsARAF3 has high homology to *H. vulgare* ARAF AXAHI and AXAHII [23], OsARAF3 was expected to cleave various arabinofuranosyl side chains, e.g., arabinoxylan and rhamnogalacturonan-I (RG-I).

The expression levels and patterns in *OsARAF1* and *OsARAF3* of leaf, stem, and root of immature seedlings (14 days old) and leaf and stem of mature plants (60 days old) were analyzed by reverse transcription (RT)-PCR expression analysis (Figure 1B). *OsARAF1* was expressed strongly in 14- and 60-day-old stems but was not detectable in 14- and 60-day-old leaves. *OsARAF3* was expressed weakly in 14- and 60-day-old leaves. These results are consistent with data retrieved from the RiceXPro (http://ricexpro.dna.affrc.go.jp; [30]). Because *OsARAF1* and *OsARAF3* are not expressed or only expressed weakly in leaves, we focused on their effects on leaves.

### *OsARAF1*-FOX and *OsARAF3*-FOX lines were ARAF overexpressors

**Figure 2:**
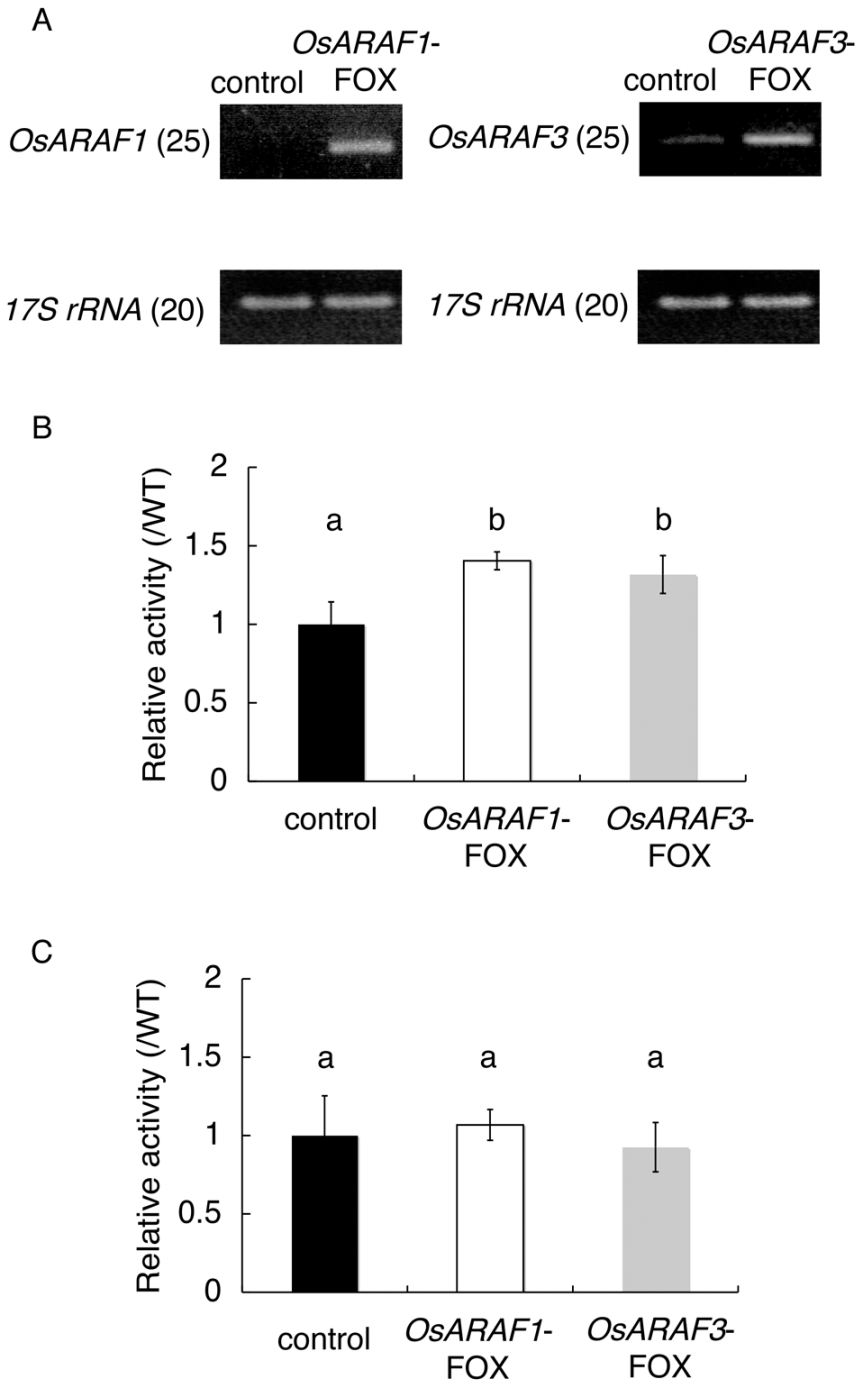
Characteristics of the *OsARAF1*-FOX and *OsARAF3*-FOX lines. (A) RT-PCR analysis of transcripts in mature leaves from the control, *OsARAF1*-FOX and *OsARAF3*-FOX lines. The levels of *OsARAF1* and *OsARAF3* transcripts were higher in each FOX line. 17S Ribosomal RNA-specific primers were used as controls. The numbers in parentheses indicate the numbers of PCR cycles. These experiments were performed at least twice with similar results. (B) Relative ARAF activities in *OsARAF1*-FOX and *OsARAF3*-FOX leaves determined using 4-nitrophenyl-α-l-arabinofuranide as a substrate. Activity is expressed as a ratio of the activity in each FOX line to that in the control leaves. Error bars indicate the SD (*n* = 3). Letters in each panel indicate significant differences at *P*<0.05 (Tukey's test). Black, white and gray columns indicate the control, *OsARAF1*-FOX and *OsARAF3*-FOX lines, respectively. (C) Relative xylosidase activities in *OsARAF1*-FOX and *OsARAF3*-FOX leaves determined using 4-nitrophenyl-β-d-xylopyranoside as a substrate. Activity is expressed as a ratio of the activity in each FOX line to that in the control leaves. Error bars indicate the SD (*n* = 3). Letters in each panel indicate significant differences at *P*<0.05 (Tukey's test). Black, white and gray columns indicate the control, *OsARAF1*-FOX and *OsARAF3*-FOX lines, respectively.

RT-PCR analysis showed that *OsARAF1* and *OsARAF3* transcripts were elevated in *OsARAF1*-FOX and *OsARAF3*-FOX, respectively (Figure 2A). ARAF activity in mature leaves, assayed using *p*-nitrophenyl arabinofuranoside as substrate, increased 1.7-fold in *OsARAF1*-FOX and *OsARAF3*-FOX compared to the control (Figure 2B and Figure S1A). Since some ARAFs have bifunctional activity for ARAF/β-d-xylosidase, we measured xylosidase activity using *p*-nitrophenyl xyloside as substrate. The xylosidase activities in *OsARAF1*-FOX and *OsARAF3*-FOX lines were almost the same as in the control (Figure 2C and Figure S1B), as predicted from the phylogenetic analysis.

**Figure 3:**
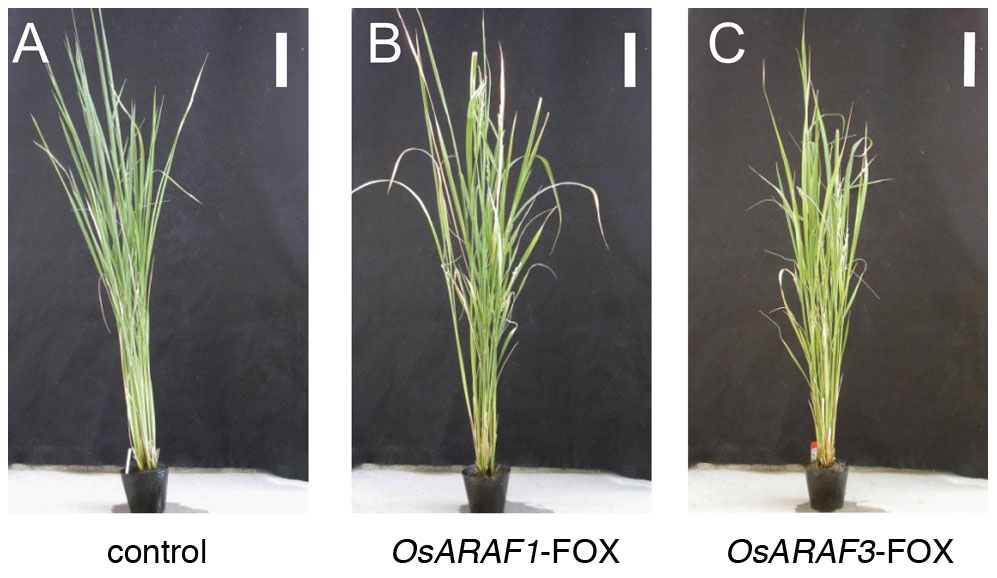
The phenotypes of the control (A), *OsARAF1*-FOX (B) and *OsARAF3*-FOX (C) in 60-day-old plant after sowing. The growth levels of FOX lines were very similar to those of the control, and all FOX lines had normal fertility. Bar  = 10 cm.

*OsARAF1*-FOX and *OsARAF3*-FOX lines, however, showed no visible phenotype (Figure 3). Vegetative parameters, plant height, stem diameter, and number of tillers, and reproductive parameters, fertility rate, number of rachis-branches, and spike length, were unchanged in *OsARAF1*-FOX and *OsARAF3*-FOX lines compared to the control (data not shown). Two FOX lines did not show high lodging.

### Effects of overexpression of ARAF on cell wall composition

We determined the glycosyl composition of cell walls prepared from mature leaves of *OsARAF1* and *OsARAF3* lines. The arabinose content in the TFA-soluble fraction, which was mainly derived from the side chain of arabinoxylan, in *OsARAF1*-FOX and *OsARAF3*-FOX lines decreased to 75.4% and 81.6% of that in the control, respectively (Table 1). The xylose content, which was derived from the main chain of arabinoxylan, also decreased in both lines. The glycosyl residue composition of cell walls isolated from leaves of the control and FOX lines was determined to establish whether overexpression of arabinofuranosidase affected the cell wall polymer content.

**Table 1:** Monosaccharide compositions of TFA-soluble fractions.

	control	*OsARAF1*-FOX	*OsARAF3*-FOX
Ara	41.9±2.6 ^a^	31.6±5.6 ^b^	33.8±6.6 ^b^
Rha	2.7±1.4 ^a^	2.5±0.9 ^a^	2.5±0.5 ^a^
Fuc	N.D.	N.D.	N.D.
Xyl	153.2±7.4 ^b^	131.6±15.9 ^a^	135.6±11.8 ^a^
Man	11.2±4.3 ^a^	9.8±3.4 ^a^	14.9±4.2 ^a^
Gal	11.8±4.3 ^a^	11.4±3.4 g	12.9±4.2 ^a^
Glc	128.3±15.2 ^a^	65.1±29.9 ^b^	90.0±18.4 ^ab^
GalA	2.3±1.0 ^a^	1.4±1.5 ^a^	2.2±1.0 ^a^
GlcA	7.3±1.9 ^a^	5.8±2.6 ^a^	9.1±3.0 ^a^
total	351.7±125 ^a^	283.6±28.3 ^b^	323.7±18.5 ^ab^

The monosaccharide compositions of alcohol-insoluble residues (AIRs) in mature leaves of the control, *OsARAF1*-FOX and *OsARAF3*-FOX were determined by GC. The values are means ± SD (*n* = 12). Different letters within the same column indicate significant differences among means (*P*<0.05) as determined by Tukey's test. N.D. means “not detected”.

**Figure 4:**
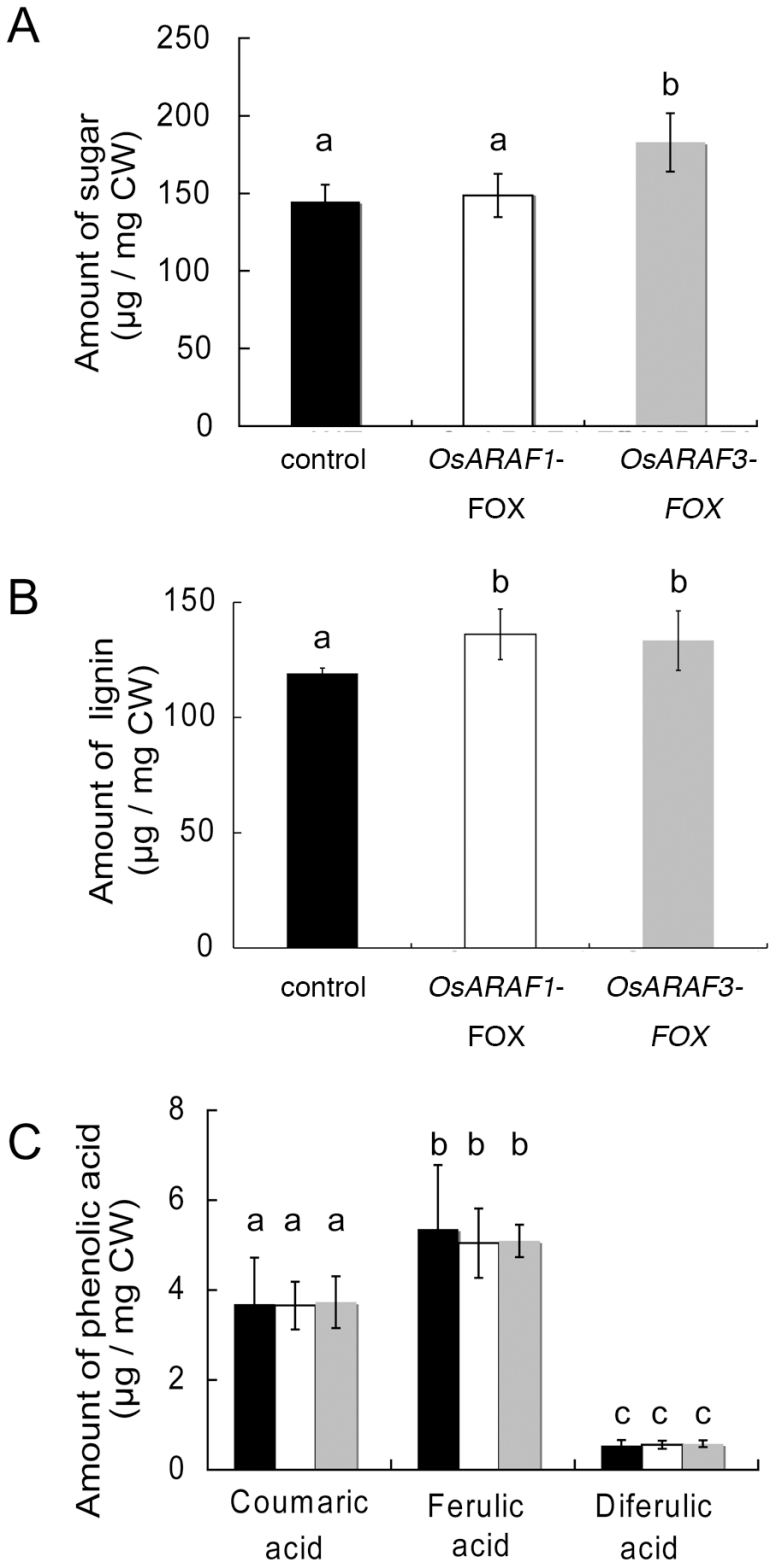
Cell wall component analysis of the control, *OsARAF1*-FOX and *OsARAF3*-FOX. The amounts of sugar in the acetic/nitric acid-insoluble fraction (A) and the amounts of lignin (B) and phenolic acids released by mild alkaline hydrolysis (C) from AIRs in the control, *OsARAF1*-FOX and *OsARAF3*-FOX lines are shown Black, white and gray symbols indicate the control, *OsARAF1*-FOX and *OsARAF3*-FOX, respectively. Error bars indicate the SD (*n* = 4). Different letters in each panel indicate significant differences at *P*<0.05 (Tukey's test).

The glucose content in the TFA-insoluble fraction in *OsARAF1*-FOX and *OsARAF3*-FOX increased to 128.2% and 134.2% that of the control, respectively (Table 2). The cellulose content was determined using the acetic/nitric acid method. The analysis revealed that the amount of sugar in the acetic/nitric acid-insoluble fraction of OsARAF3-FOX was about 120.0% that of the control (Figure 4A). The amount of lignin was slightly higher in *OsARAF1*-FOX and *OsARAF3*-FOX (Figure 4B). These results indicate that overexpression of *OsARAF1* and *OsARAF3* caused a decrease in arabinoxylan and increases in cellulose and lignin. However, the other cell wall sugars and phenolics were almost the same among the control, O*sARAF1*-FOX, and *OsARAF3*-FOX lines (Table 1, Table 2 and Figure 4C).

**Table 2:** Monosaccharide compositions of TFA-insoluble fractions.

	control	*OsARAF1*-FOX	*OsARAF3*-FOX
Ara	N.D.	N.D.	N.D.
Rha	N.D.	N.D.	N.D.
Fuc	N.D.	N.D.	N.D.
Xyl	N.D.	N.D.	N.D.
Man	27.8±5.8 ^a^	28.6±13.0 ^a^	36.4±17.4 ^a^
Gal	2.2±2.5 ^a^	4.3±11.4 ^a^	2.4±3.5 ^a^
Glc	370.3±28.1 ^a^	474.6±40.0 ^b^	497.0±32.4 ^b^
GalA	N.D.	N.D.	N.D.
GlcA	N.D.	N.D.	N.D.
total	400.3±31.3 ^a^	508.5±36.8 ^b^	535.7±32.1 ^b^

The monosaccharide compositions of alcohol-insoluble residues (AIRs) in mature leaves of the control, *OsARAF1*-FOX and *OsARAF3*-FOX were determined by GC. The values are means ± SD (*n* = 12). Different letters within the same column indicate significant differences among means (*P*<0.05) as determined by Tukey's test. N.D. means “not detected”.

### Overexpression of ARAF caused changes in localization of cell wall components

**Figure 5:**
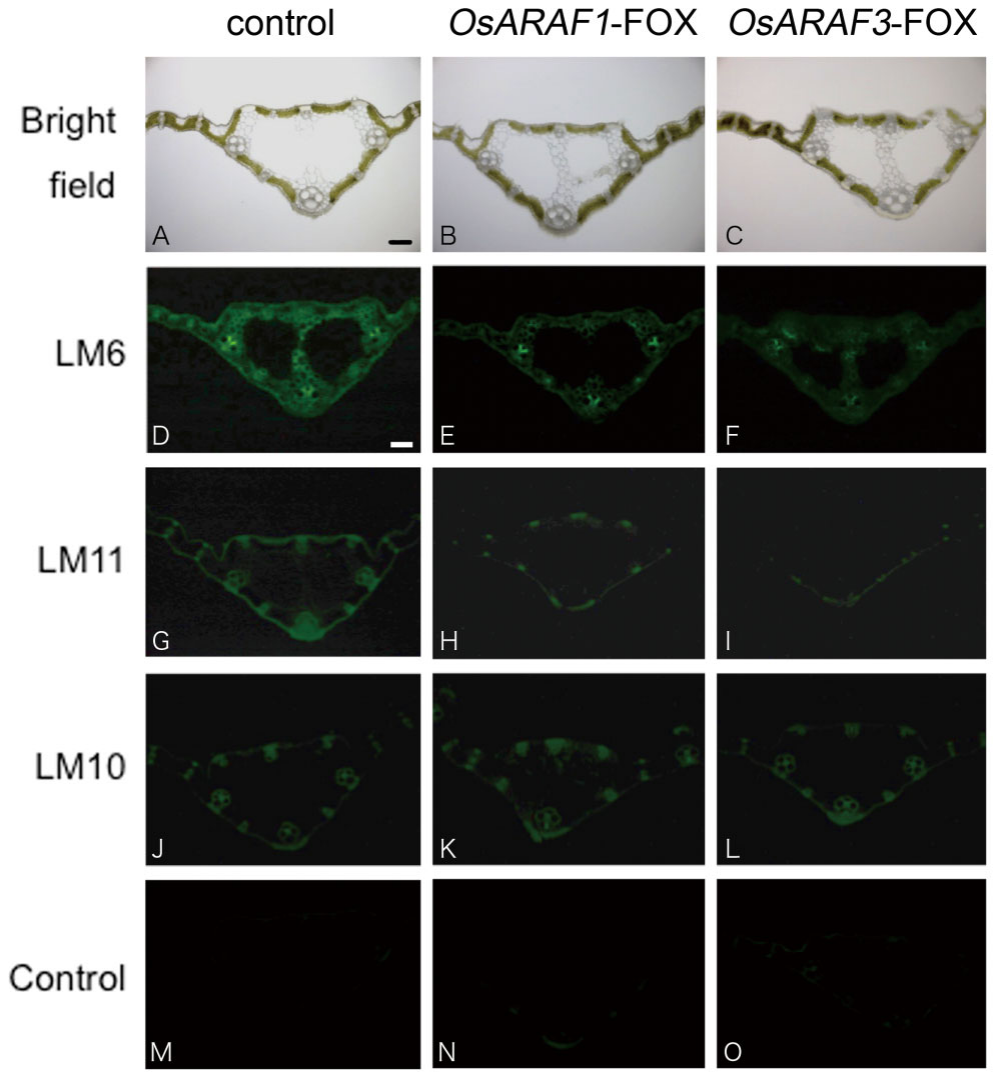
Immunofluorescent labeling of the control and FOX lines with arabinoxylan related antiibodies. Immunohistochemistry on sections of agar-embedded mature leaves of the control (D, G, J and M), *OsARAF1*-FOX (E, H, K and N) and *OsARAF3*-FOX (F, I, L and O) line were observed. The sections were labeled with the monoclonal antibodies LM6 (D–F), LM11 (G–l) and LM10 (J–L). Sections were observed under bright-field illumination (A–C). The micrographs (M–O) show the negative control performed without the first antibody step. All experiments were performed at least twice with similar results. Bars  = 100 µm.

We further determined the distribution of cell wall sugars using monoclonal antibodies against cell wall polysaccharide epitopes and Calcofluor White. The monoclonal antibody LM6 labels arabinan and arabinose side chains in arabinoxylan epitopes ([31]; University of Georgia: http://www.ccrc.uga.edu/~mao/wallmab/Antibodies/antib.htm). LM11 labeled the arabinoxylan and LM10 labeled the xylan epitope [32]. When probed with LM6, *OsARAF1* and *OsARAF3* had fewer signals in all tissues (Figure 5D–F). Moreover, LM11 stained the wall of vascular bundles and the epidermis of the control, but few signals were detected in *OsARAF1* and *OsARAF3* lines (Figure 5G–I). However, LM10 label had the same signal intensity among the control, *OsARAF1*-FOX, and *OsARAF3*-FOX lines (Figure 5J-L).

**Figure 6:**
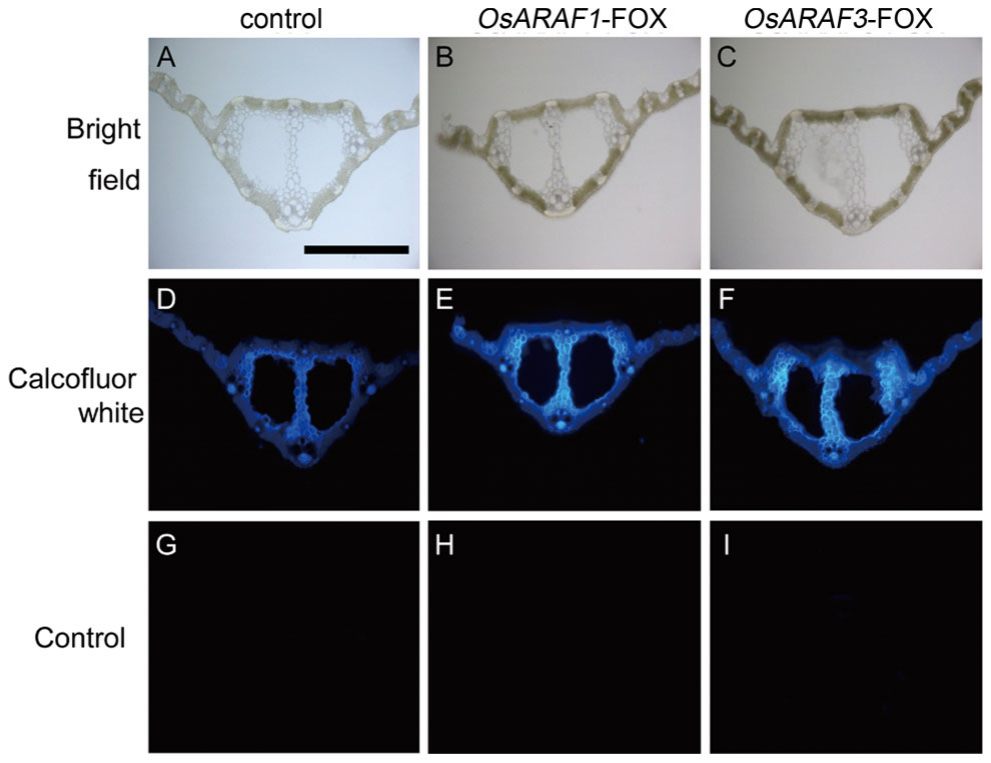
Calcofluor white staining for cellulose of mature leaves of the control and FOX lines. The section of agar-embedded mature leaves of the control (D), *OsARAF1*-FOX (E) and *OsARAF3*-FOX (F). Sections were observed under bright-field illumination (A–C). The micrographs (G–I) show negative controls performed without calcofluor white. Bars  = 500 µm. All experiments were performed at least twice with similar results.

In *OsARAF1* and *OsARAF3*, a high intensity of Calcofluor staining was observed in the midrib and phloem (Figure 6D–F). Calcofluor White is reported to stain β-1, 4-glucan (cellulose), β-1, 3-glucan (callose), and mannan [33] [34]. Because callose is present in specific cell wall components [35], and the resulting glycosyl composition showed very little mannan (Table 1 and Table 2), Calcofluor White should stain cellulose.

### Overexpression of ARAF affects the improvement of mechanical strength

**Figure 7:**
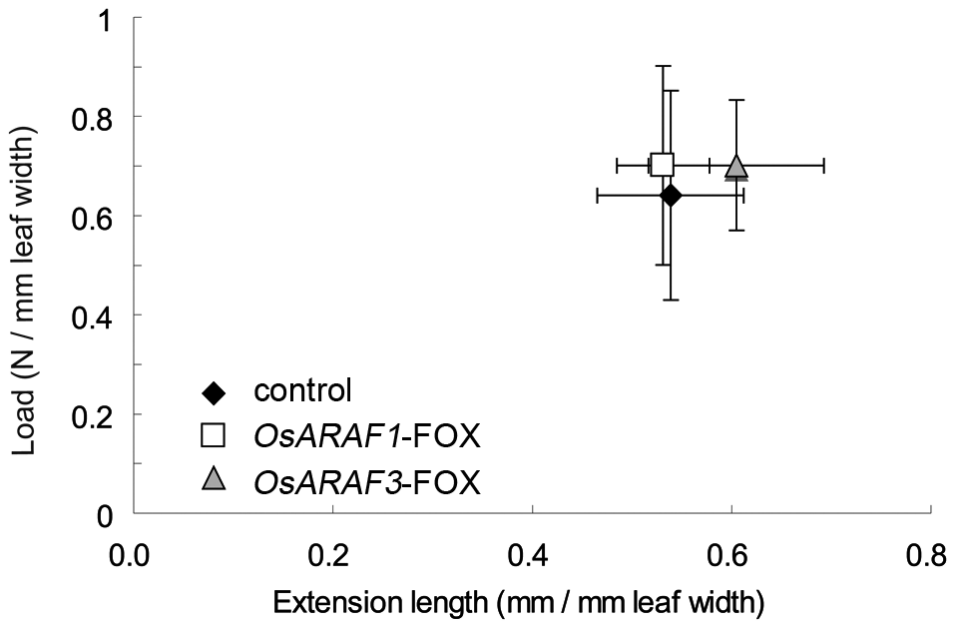
Mechanical properties of leaves of the control, *OsARAF1*-FOX and *OsARAF3*-FOX lines. The break force is expressed per leaf width. The horizontal axis shows the extension length. Black, white and gray symbols indicate the control, *OsARAF1*-FOX and *OsARAF3*-FOX, respectively. Error bars indicate the SD (*n* = 8).

Glycosyl composition analysis and histochemical observation showed that overexpression of ARAF caused a decrease in arabinose and xylose content and an increase in cellulosic glucose in the cell wall. The altered cell wall composition may influence plant mechanical properties, and thus we examined differences in mechanical properties between the control, *OsARAF1*-FOX, and *OsARAF3*-FOX leaves using the creep meter test. The breaking force and extension of leaves was similar among the control, *OsARAF1*-FOX, and *OsARAF3*-FOX. The extension length was slightly increased in *OsARAF3*-FOX (Figure 7). These results indicate that overexpression of ARAF may affect the improvement of mechanical properties of the leaf.

### Overexpression of ARAF led to an increase in saccharification efficiency

**Figure 8:**
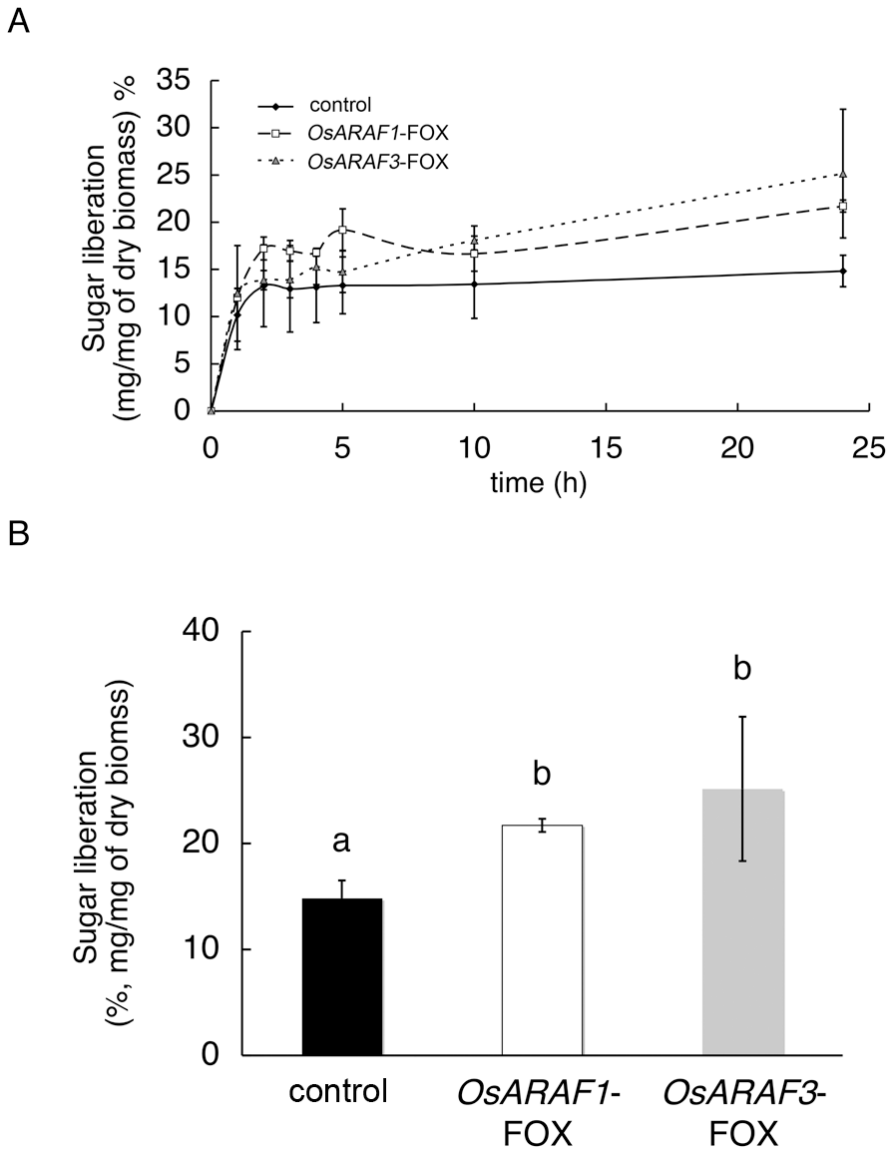
Saccharification of the control *OsARAF1*-FOX and *OsARAF3*-FOX lines in dry leaves. Time-dependent saccharification (A) and saccharification efficiency after 24 h (B) were measured. Error bars indicate the SD (*n* = 3). Black, white and gray columns indicate the control, *OsARAF1*-FOX and *OsARAF3*-FOX, respectively. Different letters in the same column indicate significant differences at *P*<0.05 (Tukey's test).

The increase in cellulosic glucose content in the cell wall of *OsARAF1*-FOX and *OsARAF3*-FOX lines implied that FOX lines produce more glucose in saccharification compared to the control. We measured sugar liberation from *OsARAF1*-FOX and *OsARAF3*-FOX mature leaves (Figure 8A and B). The rates of saccharification were almost the same for 1 h in the control, but longer reaction times led to a significantly higher saccharification rate in FOX lines than in the control, and the amount of liberated sugar after 24 h increased by 46.4% and 69.6%, respectively, compared to the control. Because the amount of starch in mature leaves accounted for only about 2% of the total sugar content (data not shown), the increase in saccharification efficiency does not appear to be influenced by starch.

## Discussion

We analyzed ARAF-overexpressed rice plants. Arabinose content in the cell walls of the two ARAF-overexpressed plants decreased to 75% and 80% of that in the control, and the amount of cellulosic glucose increased to 128.2% and 134.2% of that of control. We did not use fungal ARAF for which the exact activity against multiple substrates is known because overexpression of fungal ARAF might have resulted in lethality due to xylan degradation and inhibition of cell wall synthesis. The use of fungal cell wall hydrolases was previously shown to result in a low yield [36]; therefore, we used the endogenous arabinofuranosidases. *OsARAF1*-FOX and *OsARAF3*-FOX lines showed no morphological phenotype and some improvement of mechanical strength as the control (Figure 3 and 7). In addition, overexpression caused an increase in saccharification efficiency (Figure 8).

Arabinoxylans are major non-cellulosic polysaccharides in primary and secondary walls of grass cell walls. They are hydrogen-bonded to themselves and cellulose microfibrils to form cellulose–hemicellulose networks. Ferulic acids are ester-linked to arabinose side chains of arabinoxylan to form diferulate cross-linked arabinoxylan. In monocots, most arabinose residues exist as side chains of arabinoxylans. In secondary cell walls, ester-linked ferulic acid is incorporated into lignin to form an arabinoxylan–lignin complex [9]. Hence, arabinose residues are thought to be the base point for cross-linking between arabinoxylan and lignin [37]. In growing tissues, arabinoxylan content in cell walls increases slowly and arabinoxylans are highly branched with arabinosyl residues and are removed during growth [38] [39].

ARAFs are defined as enzymes that catalyze the hydrolysis of terminal, nonreducing α-l-arabinofuranoside residues. However, several enzymes in family 51 are capable of hydrolyzing both l-Ara and d-Xyl from a variety of substrates in vitro, and therefore may be considered as bifunctional ARAF/β-d-xylosidase enzymes. For example, barley ARA-I, *Arabidopsis* XYL3 and ARAF1, and alfalfa MsXyl1 have bifunctional enzyme activity [25] [26] [40] [41]. However, our results showed that OsARAF1 and OsARAF3 may have only ARAF activity and no xylosidase activity (Figure 2B and C). And, lignin complexes with arabinoxylan are mediated by ferulic acid [9]. In *OsARAF1*-FOX and *OsARAF3*-FOX, the amounts of ferulic acid were similar to that in the control. These results suggest that OsARAF1 and OsARAF3 do not possess the ability to cleave the linkage of the arabinose residue to ferulic acid. Expression of OsARAF1 and OsARAF3 was higher in growing organs and lower in mature organs (Figure 1B). These results indicate that arabinoxylans are turned over during cell wall development in growing organs.

Arabinose residues are a quantifiably important constituent in various cell wall components, not only arabinoxylan but pectin RG-I and glycoprotein [2] [42]. Glycosyl composition analysis of cell walls and immunohistochemistry showed that arabinosyl residues in arabinoxylans decreased in *OsARAF1*-FOX and *OsARAF3*-FOX lines. The xylan in *OsARAF1*-FOX and *OsARAF3*-FOX also decreased in the hemicellulose fraction (Table 1). This result suggests that the xylan main chain decreases by reducing the side chain arabinose in FOX lines. The ratios of arabinose to xylose in *OsARAF1*-FOX and *OsARAF3*-FOX were 1∶3, the same as that in the control (Table 1). These results indicate that a change occurred in the quantity rather than the structure of arabinoxylan in the FOX lines as compared to the control. The amounts of arabinose side chains may influence the xylan backbone extension, and arabinose residues may be important in the formation of the cell wall network. On the other hand, overexpression of *AtARAF1* leads to increases in the secondary cell wall, especially xylan [43]. In any case, arabinofuranosidase overexpression may cause modification of the secondary cell wall.

Neither *OsARAF1*-FOX nor Os*ARAR3*-FOX affected plant growth (Figure 3). The increase in cellulose may compensate for the reduction in arabinoxylan (Table 1, Table 2 and Figure 6). Additionally, amount of crystalic cellulose in *OsARAF3*-FOX increased compared to the control (Figure 4A). Staining with Calcofluor White increased in the midrib and phloem of FOX lines, which is associated with the increased mechanical strength (Figure 6D–F and Figure 7). From these results, it appears that rice might maintain plant body strength by maintaining a balance between cellulose and hemicellulose. In fact, *OsARAF1*-FOX and *OsARAR3*-FOX lines showed slightly increase of mechanical strength compared to the control (Figure 7). In dicots, xyloglucan is hydrogen-bonded to cellulose microfibrils. In xylogulcanase-overexpressed poplar, the amount of cellulose increased [44] and led to the generation of abnormal tensile stress. In this study, ARAF-overexpressed rice had a lower arabinoxylan content, and so it was expected to have a similar phenotype; however, no influence on growth occurred (Figure 3). Unlike dicots, rice has interspersed vascular bundles, which have less influence on retaining the strength of the plant body, so an increase of cellulose may have a positive effect on growth.

*OsARAF1*-FOX and *OsARAF3*-FOX lines had increased saccharification efficiency (Figure 8A and B). In industrial processes, plant biomass is treated with hemicellulase and cellulase because cellulose covers the cell wall matrix. We expected that the increased saccharification efficiency of FOX lines would depend on decreases in hemicellulose and substituted xylan [45]. The saccharification efficiency after 24 h reaction increased in FOX lines (Figure 8A), which may have been caused not only by an increase in accessibility of the cellulose due to a decrease in hemicelluloses but also an increase in cellulose content. In cell walls, glucose is contained in cellulose and β-1,3-1,4-glucan. The hydrolysis of β-1,3-1,4-glucan is accelerated by growth regulators; in mature leaves, the β-1,3-1,4-glucan level is low [2]. The amount of lignin increased slightly in *OsARAF1*-FOX and *OsARAF3*-FOX lines, but this didn’t influence saccharification efficiency. Lignin complexes with arabinoxylan and is mediated by ferulic acid [9]. In this study, the decrease in arabinoxylan had little effect on cell wall network assembly and growth. The small change in cell wall components led to higher cellulose content in the plant but had no effect on growth. And, in previous studies, few examples of increases in cellulose content in cell walls were reported. Hence, our report provides an important contribution to the study of cell wall biosynthesis and future technology. The modification cell walls in rice through ARAF overexpression improves saccharification efficiency, and these FOX rice lines could prove useful for producing cellulosic bioethanol.

## Supporting Information

Figure S1 **Enzyme activity in WT,** ***OsARAF1*** **-FOX and** ***OsARAF3*** **-FOX.** (A) ARAF activity using 4-nitrophenyl-α-l-arabinofuranide as substrate in WT, *OsARAF1*-FOX and *OsARAF3*-FOX leaves. (B) Xylosidase activity using 4-nitrophenyl-β-D-xylopyranoside as substrate in WT, *OsARAF1*-FOX and *OsARAF3*-FOX leaves. Black, white, and gray columns indicate the WT, *OsARAF1*-FOX, and *OsARAF3*-FOX lines, respectively. Black, white, and gray columns indicate WT, *OsARAF1*-FOX, and *OsARAF3*-FOX, respectively.
(PDF)
