# Supplementary material for: Increase in Cellulose Accumulation and Improvement of Saccharification by Overexpression of Arabinofuranosidase in Rice
Source: PLoS One. 2013 Nov 4;8(11):e78269. doi: 10.1371/journal.pone.0078269 (PMC3817243; doi:10.1371/journal.pone.0078269)
Supplement: Figure S1 — Enzyme activity in WT, OsARAF1 -FOX and OsARAF3 -FOX. (A) ARAF activity using 4-nitrophenyl-α-l-arabinofuranide as substrate in WT, OsARAF1-FOX and OsARAF3-FOX leaves. (B) Xylosidase activity using 4-nitrophenyl-β-D-xylopyranoside as substrate in WT, OsARAF1-FOX and OsARAF3-FOX leaves. Black, white, and gray columns indicate the WT, OsARAF1-FOX, and OsARAF3-FOX lines, respectively. Black, white, and gray columns indicate WT, OsARAF1-FOX, and OsARAF3-FOX, respectively. (PDF) [file pone.0078269.s001.pdf]

Increase in cellulose accumulation and improvement of saccharification by overexpression of arabinofuranosidase in rice

Minako Sumiyoshi, Atsuko Nakamura, Hidemitsu Nakamura, Makoto Hakata, Hiroaki Ichikawa, Hirohiko Hirochika, Tadashi Ishii, Shinobu Satoh and Hiroaki Iwai

A

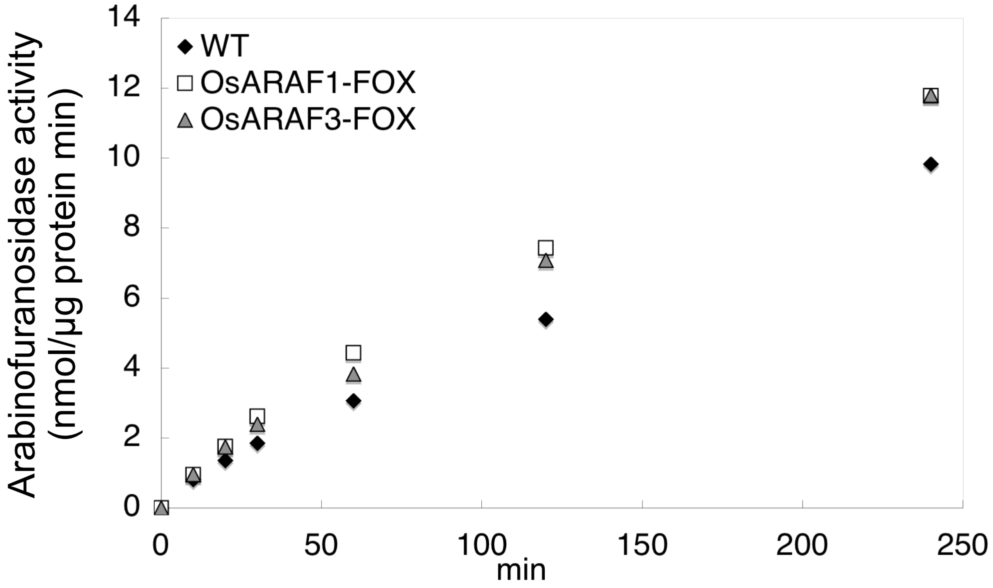

B

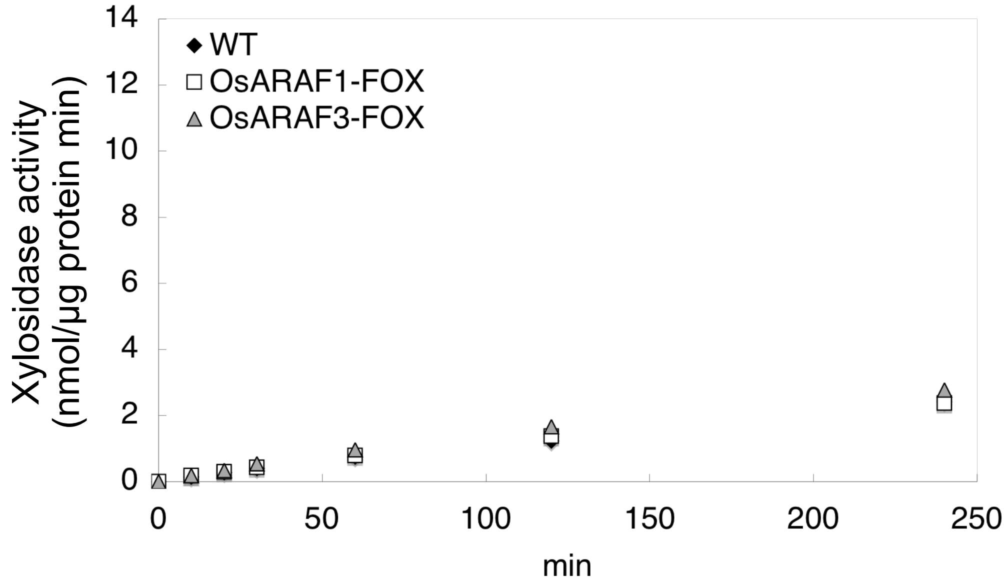

Figure S1 Enzyme activity in WT, *OsARAF1*-FOX and *OsARAF3*-FOX.
